# Transforming Schottky to Ohmic Contacts via Ultrahigh-Vacuum Engineered Interfacial Alloying

**DOI:** 10.1021/acsami.5c21524

**Published:** 2025-12-05

**Authors:** Masoud Ebrahimzadeh, Perttu Piispanen, Sari Granroth, Mikko Miettinen, Ilari Angervo, Hanchen Liu, Markus Otsus, Risto Punkkinen, Marko Punkkinen, Ville Vähänissi, Kalevi Kokko, Petriina Paturi, Kaupo Kukli, Hele Savin, Pekka Laukkanen

**Affiliations:** † Department of Physics and Astronomy, 8058 University of Turku, Turku FI-20014, Finland; ‡ Department of Electronics and Nanoengineering, 174277 Aalto University, Espoo FI-02150, Finland; § Institute of Physics, University of Tartu, Tartu EE-50411, Estonia

**Keywords:** ohmic contact, n-type Ge, surface doping, lift-off processing, ultrahigh vacuum

## Abstract

Low-resistive Ohmic contacts are needed in most microelectronics and photonics devices to connect a device to the electric circuit. Manufacturing of Ohmic contacts typically requires the doping of a semiconductor surface region as n-type or p-type (i.e., electron- or hole-doped, respectively). This task has, however, become challenging when the doping needs to be controlled with nanometer or even atomic level precision at lowered processing temperatures. In this work, we demonstrate a low-temperature method to tackle this contact manufacturing challenge using ultrathin antimony (Sb) doped germanium (Ge) nanolayers. We have integrated the method with the common lift-off processing to make Ohmic nickel (Ni) contacts on low-doped n-type Ge and Si substrates and on semi-insulating GaAs, which initially show the Schottky contacts. A proper combination of wet chemical cleaning plus depositing Sb and Ge atomic layers on the substrates, kept at room temperature, in a very clean environment of ultrahigh vacuum before the Ni-film deposition and postmetallization heating changes the Schottky contacts to Ohmic ones. Complementary methods are used to probe the physicochemical properties of interfaces during the manufacturing process to clarify the mechanisms behind the Ohmic-contact formation.

## Graphical abstract

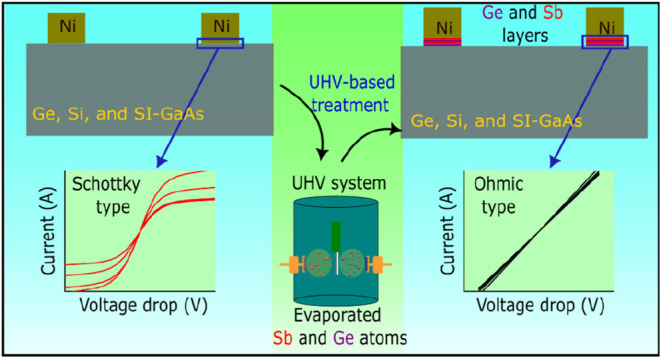

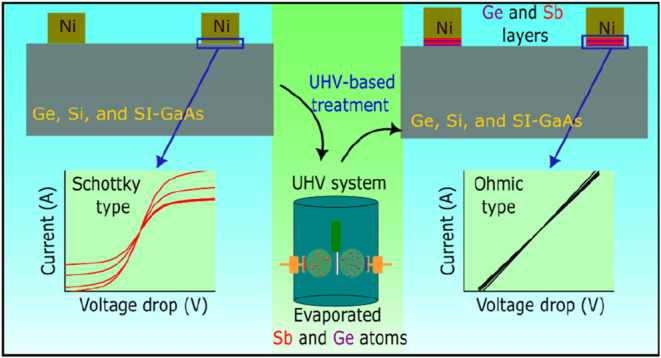

## 1 Introduction

Durable and low-resistive Ohmic metal–semiconductor contacts with minimized carrier recombination have been intensively investigated and developed for several decades (e.g., refs 1–20 ) because these contacts play a key role in the final performance of various semiconductor devices, for example, affecting the energy efficiency and usage time of solar cells and transistors. Increasing the doping concentration (n-type or p-type) at the semiconductor surface areas is a prerequisite to preparing Ohmic metal contacts. With decreasing semiconductor device dimensions and increasing devices’ integration density, the need for methods to manufacture highly doped and ultrashallow semiconductor areas with atomically sharp interfaces has increased significantly. The traditional methods to modify the semiconductor doping concentration, i.e., ion implantation plus postheating or diffusion of doping elements at elevated temperatures, do not perform properly when nanometer or even atomic-level control of dimensions of the doped areas is required and when the doping needs to be done in later stages of the device processing line (e.g., at back-end-of-line stages) at lowered processing temperatures.

Monolayer doping of semiconductor surfaces has been one of the most intensively studied methods to overcome the doping challenges with smaller and smaller device structures.^7,9,10,12,14,15,17^ Briefly, in this method, a layer of doping atom-containing organic molecules (e.g., phosphonate and phosphonic on Si) is deposited on a semiconductor surface. This surface is still covered by an insulator film like SiO_2_. Then, the whole stack is postheated at elevated temperatures (often >600 °C) to incorporate doping atoms (e.g., phosphorus into Si) into the semiconductor lattice sites via temperature-induced interdiffusion of atoms at the semiconductor interface. This novel method is simple and scalable toward industrial use, but it also has some weaknesses, as the high-temperature postheating is not necessarily possible if a processed device includes a part(s) made of a material that degrades during the heating. Use of organic molecules also increases the risk for carbon contamination and degradation of carrier transport properties.^17,21^ Furthermore, an emergent method is based on using controlled laser light exposures to anneal, for example, selective areas locally.^22–24^ However, there is still room for alternative solutions to reduce contact-induced losses in various devices.

Selective-area epitaxial regrowth of highly doped semiconductor crystals (e.g., Si_*x*_Ge_1–*x*_, Ge, Ge_*x*_Sn_1–*x*_) combined with an opportunity for atomic-layer thickness control is another elegant method^25–32^ which has gained increasing interest recently because this method enables one to control the doping near the source and drain areas of the three-dimensionally structured FinFET devices.^31,32^ Typically, the chemical vapor deposition of selective-area semiconductors is done at elevated temperatures. However, the growth temperature of boron-doped Ge and Ge_*x*_Sn_1–*x*_ has recently been decreased below 350 °C with promising results.^28^

Use of Ge increases in cutting-edge semiconductor technologies such as high-speed and low-power integrated circuits and infrared detectors.^33–39^ The preparation of Ohmic metal contacts at n-type Ge has been more challenging than for p-type Ge because the surface Fermi level becomes pinned or locked near the valence-band maximum (Figure 1), leading to the Schottky-type band bending at n-Ge.^40–45^ Detailed reason(s) for the strong Fermi-level pinning at n-Ge interfaces are still unknown. To obtain an Ohmic-type linear current–voltage behavior and to decrease the contact resistivity at metal/n-Ge interfaces, two main approaches have been employed: (i) insertion of an ultrathin insulator layer (e.g., ZnO and TiO_2_) between n-Ge and metal, and (ii) increasing the n-type doping concentration at the Ge surface.^46–49^ The former decreases the Schottky-type band bending, whereas the latter provides a narrowed depletion region for electron tunneling, which boosts current flow at the interface (Figure 1). To overcome the challenges with the traditional doping methods, the monolayer doping of Ge^14–16^ and the selective-area regrowth^27,30,31^ have been studied with promising results. Moreover, depositing Sb metal layers on Ge and then heating the material by laser illumination has provided diffusion of Sb into the Ge substrate at 10–20 nm depth,^18^ where lasers provide a new controlled method to heat selected areas of the material.^50,51^

**Figure 1:**
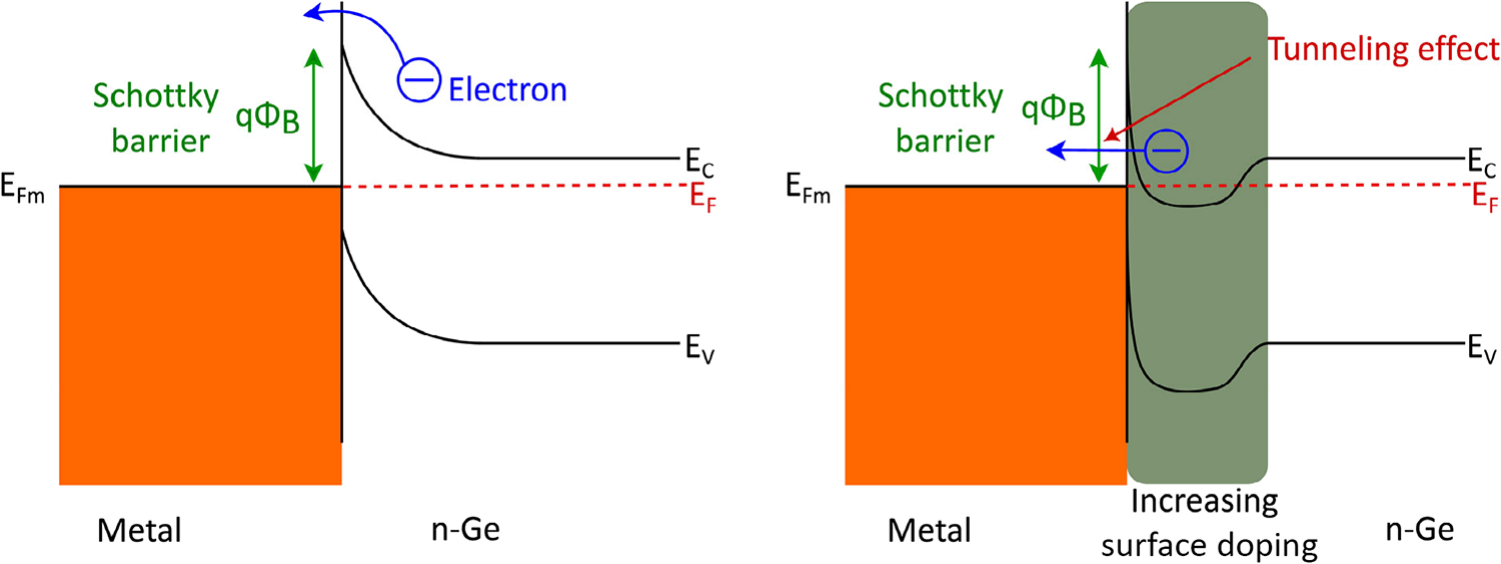
Schematic illustration of the effect of surface doping on the band diagram of a Schottky-type n-Ge contact. Left-side situation causes the Schottky-type current–voltage curve, while the right-hand situation provides Ohmic-like current–voltage lines due to an ultrathin tunneling barrier. *E*_C_, *E*_V_, *E*_F_, and *E*_Fm_ represent the energies of the conduction band, valence band, Fermi level, and metal Fermi level, respectively.

Another crucial factor behind the Ohmic-contact formation is the cleaning of a semiconductor surface before depositing a metal film(s) because the physicochemical properties (e.g., oxidation state and crystalline order) of the starting surfaces affect the overgrown metal interfaces. Wet chemical treatments have been the main cleaning method also for Ge surfaces^52^ like for most semiconductors in general. A problem with the wet chemical cleaning is that the resulted surface structure is quite disordered, although it removes most of the surface oxides and contaminants from the semiconductor surfaces.^53^ It is surprisingly difficult to prepare and maintain an atomically smooth and well-ordered semiconductor surface. Molecules from the environment are quickly readsorbed on a cleaned surface, even if the metal (or film) deposition is done in a high-vacuum background (1 × 10^–8^ to 1 × 10^–4^ mbar) of an instrument. A clear benefit has been found when a metal film growth is done in a very clean environment of ultrahigh vacuum (UHV, 1 × 10^–12^ to 1 × 10^–8^ mbar) where surface interactions with background gases are reduced.^54–57^

In this work, we demonstrate a low-temperature method to increase the surface doping level at low-doped n-Ge by growing ultrathin (≤10 nm) Sb-doped Ge films, which change initial nickel (Ni) Schottky contacts to the Ohmic ones. We have integrated this method with common lift-off processing to modify the surface properties in selected areas. The presented results on a low-doped Si substrate and semi-insulating GaAs (SI-GaAs) indicate that the Sb-doped Ge films might also be used on other semiconductors to modify their metal contact interfaces. We have utilized the UHV environment to engineer cleanliness and the surface doping level. The review is organized as follows. First, we have studied cleanliness and surface doping of n-Ge substrates, and after that, we transferred the results on Si and SI-GaAs to prepare Ohmic contacts. Complementary measurements and simulations are performed to understand physicochemical properties at the interfaces.

## 2 Method Section

### 2.1 Material Preparation

Pieces of low-doped n-type Ge and Si, as well as of SI-GaAs wafers, 6 × 12 mm^2^, were used in experiments. The phosphorus dopant concentration of these Ge wafers ranged from 5 × 10^13^ to 1 × 10^14^ cm^–3^, while for low-doped n-Si, the phosphorus concentration was 5 × 10^14^ to 1 × 10^15^ cm^–3^. The SI-GaAs resistivity was in the range of 10^8^ Ω cm. Both Ge and GaAs samples were chemically pretreated using a HCl-based solution diluted with isopropanol (IPA) at a ratio of 3:1. Then, the Ge and SI-GaAs pieces were immersed in IPA for 1 min and dried by using N_2_. Si surfaces were chemically cleaned using HF (5%) solution, followed by rinsing in deionized water (DW) and N_2_ drying. Some separate highly Sb-doped n^+^-Ge wafer pieces (1 × 10^17^ cm^–3^) were also used. Two basic procedures were tested to process patterns of Ni/n-Ge contacts: (i) chemical etching of metal and (ii) lift-off lithography:

(i) After the wet chemical cleaning, samples were air-transported (almost 15 min) for the deposition of a 100 nm Ni layer to cover the whole surface. The metal deposition was carried out using a BalTec Med 020 sputtering device. During the Ni depositions, substrates were at room temperature. Metal-pad patterns with varying pad spacings for the transfer length measurement (TLM) structures were fabricated using photolithography and HCl-based (12%) etching to remove Ni between the pads.
(ii) The TLM structure was fabricated using a DILASE-250 tabletop laser to create a hole pattern in photoresist. Next, a 100 nm film of Ni was deposited onto the samples. Excess Ni between the pads was then removed by removing the photoresist with acetone, followed by thorough cleaning with IPA to decrease the amount of contaminants.

After careful characterization (Table S1 and Figure S1) of the Ni/n-Ge contacts prepared by the metal etching approach (i), we excluded this method in our experiments because we found that a surprisingly strong NiGe_*x*_ alloy was formed at the n-Ge surfaces even at room temperature deposition. In other words, we could not remove a NiGe_*x*_ alloy between the pads using wet chemistry, which caused a low-resistive parallel surface channel between the pads (Figure 2a), making the contact resistivity determination challenging. We confirmed that the lift-off samples did not include the NiGe_*x*_ alloy surface channel between the contact pads. Thus, we used lift-off manufacturing (ii) to test the effects of Sb and Ge nanolayers on the contact resistivity.

**Figure 2:**
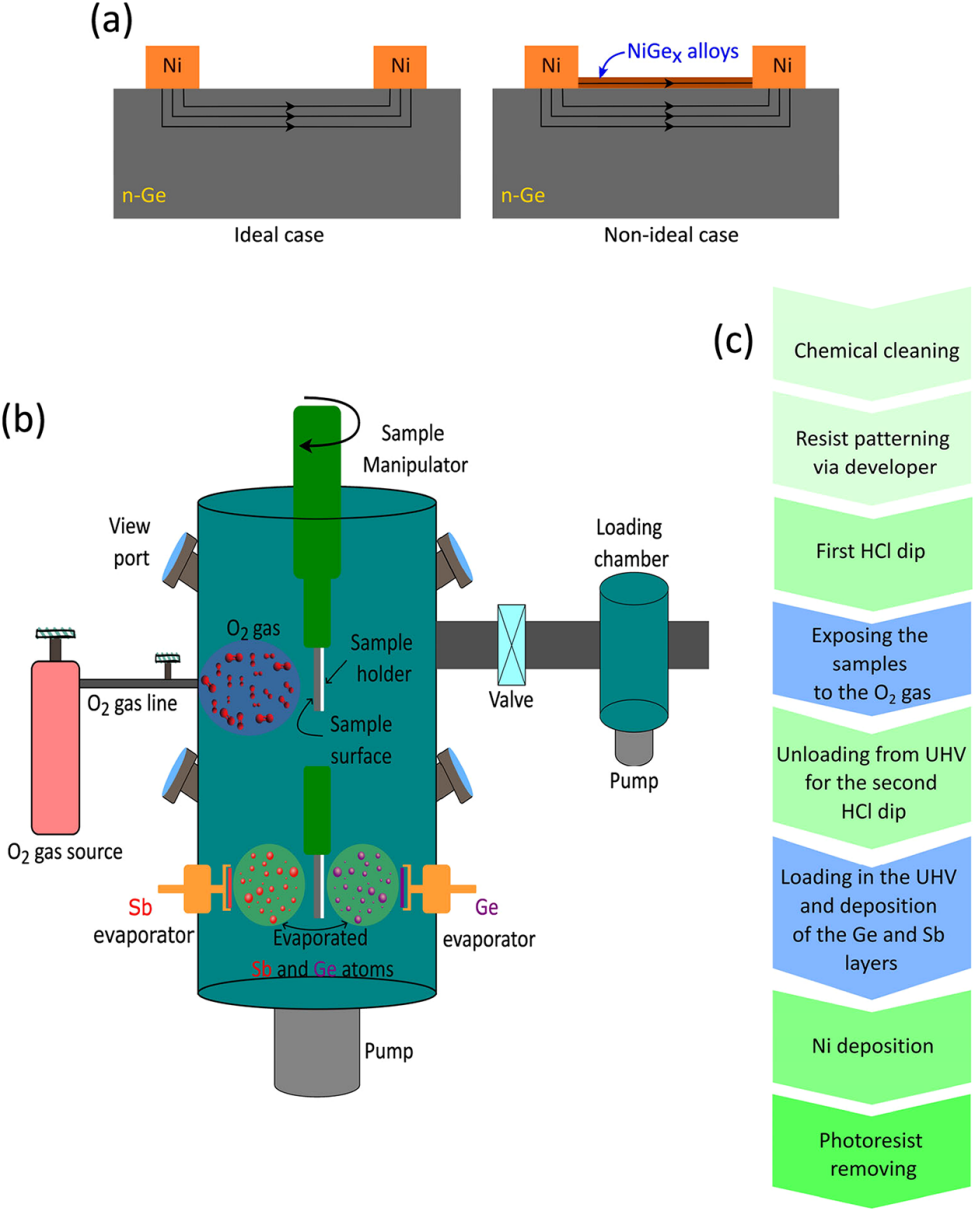
(a) Left: a proper sample type for the TLM measurement to determine the contact resistivity. Right: we found that NiGe_*x*_ alloy readily formed at n-Ge, although Ni deposition was done at room temperature. Thus, we excluded the metal etching method and used the lift-off process in this work. (b) Schematic representation of the UHV chamber where atomic layers of Sb and Ge were deposited. During intentional oxidations, O_2_ partial pressure was 1 × 10^–5^ mbar. (c) Part of the device processing line, which we focused on in this work: stages with blue color were done in UHV; details varied between samples, as summarized in the tables.

Modifications of n-Ge, n-Si, and SI-GaAs surfaces were performed in the preparation unit (with a base pressure of 1 × 10^–8^ mbar) of a UHV multichamber system (Figure 2b). Additionally, some characterizations were performed in an in situ manner in the analysis chamber of the same UHV system. Depositions of Sb and Ge atomic layers were carried out using thin tantalum envelopes that wrapped Sb or Ge pieces and were heated by direct current. The thickness of the Sb and Ge layers was determined using the Beer–Lambert Law.^58^ Some Ge surfaces were intentionally oxidized in the UHV chamber by feeding the O_2_ gas via a leak valve. Subsequently, the oxidized surface was still re-etched in the HCl solution. A target was here to reduce the defect amount by a cycle of oxidation and oxide removal from the pad areas before the Ni deposition. The workflow is depicted in Figure 2c, with detailed information available in the tables presented in the results sections. The metal-contact samples were still postheated in the UHV system step by step.

### 2.2 Surface and Electrical Characterization

To study the properties of the surfaces and interfaces, complementary techniques were employed: X-ray photoelectron spectroscopy (XPS) with a monochromatized (Al–Kα) Thermo Scientific Nexsa system, scanning tunneling microscopy (STM, Omicron Scala), and low-energy electron diffraction (LEED). The structural properties of the films were analyzed through X-ray diffraction (XRD) using an Empyrean diffractometer equipped with a 5-axis goniometer and an Empyrean Cu LFF HR tube. The X-rays were monochromatized into Cu Kα components. Scanning Electron Microscopy (SEM) analysis was conducted using a Thermo Fisher Scientific APREO field emission SEM (FE-SEM), equipped with an EDS detector, to assess the surface regions between metal pads. Some samples were further analyzed using a Cs-corrected Titan Themis 200 microscope alongside an FEI Super-X EDS system. The lamellae for STEM observations were made using a Helios Nanolab 600 Scanning Electron Microscope (SEM) and a Focused Ion beam system. Among the techniques mentioned above, STM and LEED are *in situ*, while others are *ex situ*. Transfer length measurements (TLM) were used to determine the contact resistivity (ρ_c_) by using an HP4145B semiconductor parameter analyzer. The analyzer is linked to the Rucker & Kolls 666 four-point probe needle probing stage.

Work functions were calculated by using the density functional theory program, the VASP program. Nine atomic layer slabs were used. The (100) surface area was (4 × 4) for Ge [c(4 × 2) reconstruction] and Sb-doped Ge and (1 × 1) for Ni.

## 3 Results and Discussion

A key aspect of the lift-off process is patterning of a resist layer on a semiconductor surface before metallization. Removing the resist from the areas of metal contacts causes exposure of these semiconductor areas to atmospheric conditions in transferring the sample to a metal-deposition instrument. Our target here is to study a protocol that can be combined with that stage of device processing. We have investigated the effects of (i) intentional surface oxidation with O_2_ and (ii) incorporation of a nanolayer stack of Sb and Ge on the substrate before Ni-film deposition.

### 3.1 Effects of Intentional Oxidation on Low- and High-Doped n-Type Ge

One challenge in the lift-off process is ensuring that the resist layer remains intact during any intermediate cleaning or layer deposition step. The resist layer does not tolerate temperatures much higher than 100 °C. To decrease the density of surface defects at the exposed Ge surface areas in the patterned resist before the Ni deposition, the following approach was used: a cycle of dilute HCl immersion and UHV-based O_2_ exposure. The current–voltage (*I–V*) measurements (Figure S2) for both low- and high-doped n-type Ge substrates show that the intentional oxidation + HCl etching reduces the ρ_c_ (Table 1). This reduction is pronounced for high-doped n-Ge, where an Ohmic contact is achieved through postheating at 350 °C, resulting in ρ_c_ of 1.8 × 10^–3^ Ω cm^2^. This is because the highly doped n-type Ge substrate facilitates Ohmic-contact formation.

**Table 1:** Effects of Surface Cleaning on Ni/n-Ge Contacts Prepared by the Lift-Off Process on Both Low-Doped and High-Doped n-Ge Substrates^a^

									contact resistivity (Ω cm^2^)			
doping level	sample	chemical cleaning of n-Ge	lift-off lithography	HCl dip (s)	O_2_ exposure in UHV (min)	HCl dip (s)	Ni film thickness (nm)	photoresist remover	as-ready	postheat at 350 °C for 60 min	postheat at 500 °C for 30 min	postheat at 600 °C for 30 min
low doped	G5	HCl (9 M) for 5 min + IPA dip for 1 min + dried with N_2_	√				100	acetone	Sch.	Sch.	Sch.	Sch.
	G6			90					Sch.	Sch.	Sch.	0.83
	G7			90	60	90			Sch.	Sch.	Sch.	0.74
high doped	G8			90					Sch.	Sch.		
	G9			90	60	90			Sch.	1.8 × 10^–3^		

a (Schottky is denoted by Sch.).

To clarify the effects of the intentional surface oxidation of Ge, XPS measurements were done on highly doped n-type Ge as a function of the exposure to O_2_ and HCl-based etching. Figure 3 indicates the presence of a GeO_*x*_-induced shoulder at 1220 eV on the surface after removing the photoresist from the contact areas, likely resulting from the exposure of the surface to air in transferring the sample (about 15 min in air). Although the HCl immersion decreases this shoulder, a nonzero intensity bump appears around 1225 eV, indicating an increase in GeO_2_ surprisingly (Figure 3; inset). A subsequent surface exposure to O_2_ gas in the UHV chamber, plus the HCl immersion, decreased these oxide-related features. The LEED pattern (Figure 3; inset) from the surface after the second HCl dip shows (1 × 1) diffraction spots in agreement with the XPS finding that the amount of Ge oxides decreases. No LEED pattern was observed from this surface, which was treated by the resist lift-off process, before the second HCl immersion. This indicated that the thickness of a highly disordered Ge surface layer was at least 0.5 nm in the exposed areas, where the resist was removed, until the second HCl immersion. XPS measurements in Figure S3 indicated that the C 1s intensity remained largely unchanged throughout these treatments. These results imply that decreasing the disordered Ge layer thickness before the Ni deposition contributes to the Ohmic-contact formation (Table 1).

**Figure 3:**
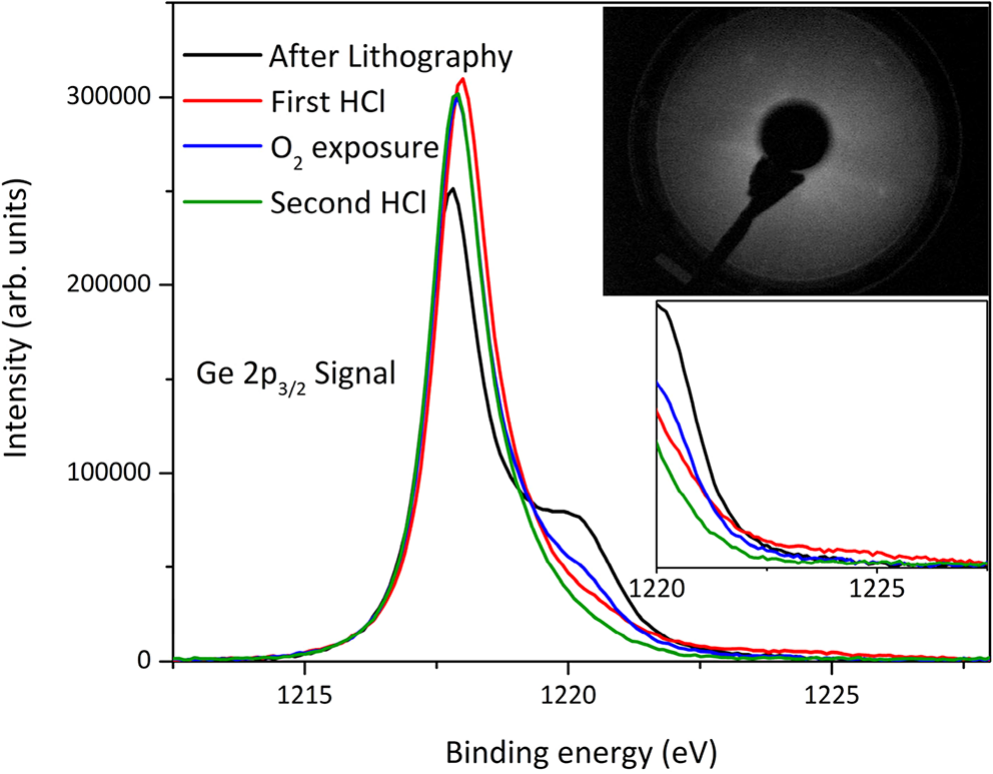
Changes in the Ge 2p_3/2_ peak as a function of O_2_ exposure and HCl etching. The inset shows LEED after the cleaning protocol: HCl + O_2_ exposure + HCl.

### 3.2 Effects of Sb-Doped Ge Nanofilm on Low-Doped n-Type Ge Substrate

As Table 1 exemplifies, the manufacturing of Ohmic contacts on low-doped n-Ge is difficult, as expected. The results shown in Section 3.3 for low-doped n-Si support the challenge. In order to reveal the potential of an alternative approach, we have studied the effects of Sb and Ge nanolayers deposited on the cleaned n-Ge substrate surface. That is the cycle of HCl + O_2_ exposure + HCl cleaning was done before growing Sb or Ge layers in the UHV chamber (Figure 2), keeping the substrate with the resist pattern at room temperature. Table 2 provides a summary of the observed contact resistivities. All as-prepared contacts were initially measured and subsequently subjected to postheating after the Ni deposition at temperatures ranging step by step from 350 to 600 °C in the UHV environment. The incorporation of a mere Sb layer (1–3 nm) on the low-doped n-Ge substrate resulted in the formation of an Ohmic contact at a lower postheating temperature (500 °C), compared to the reference Ni/n-Ge contact without the Sb layer. We did not find any clear change as a function of the Sb-layer thickness between 1 and 3 nm.

**Table 2:** Properties of Ni/Sb/n-Ge and Ni/Sb-Doped Ge/n-Ge Contact Interfaces Prepared by the Lift-Off Process on the Low-Doped n-Ge(100) Substrate

											contact resistivity (Ω cm^2^)			
sample	chemical cleaning of n-Ge	lift-off lithography	HCl dip (s)	O_2_ exposure in UHV (min)	HCl dip (s)	Sb deposition (nm)	Ge deposition (nm)	Sb deposition (nm)	Ni film thickness (nm)	photoresist remover	as-ready	postheat at 350 °C for 60 min	postheat at 500 °C for 30 min	postheat at 600 °C for 30 min
G10	HCl (9 M) for 5 min + IPA dip for 1 min + dried with N_2_	√	90	60	90	1			100	acetone	Sch.	Sch.	0.55	0.92
G11						2					Sch.	Sch.	0.50	0.70
G12						3					Sch.	Sch.	0.51	
G13						2	4	2			Sch.	2 × 10^–2^	1.2 × 10^–3^	

When a stack of Sb and Ge nanolayers (2 nm Sb + 4 nm Ge + 2 nm Sb) was deposited on the low-doped n-Ge substrate with the resist pattern at room temperature before Ni, the Ohmic contact with ρ_c_ of 2 × 10^–2^ Ω cm^2^ was obtained using the lowered postheating at 350 °C. Increasing the postheating temperature to 500 °C decreased ρ_c_ to 1.2 × 10^–3^ Ω cm^2^ (Sample G13, Table 2). In Table 3, we have presented a benchmarking comparison with the methods and results found in the literature.

**Table 3:** Comparison to Selected Methods and Resistivities Found for n-Type Ge Contacts

**substrate**	**year**	**substrate doping**[cm^**–3**^ **]**	**activation annealing**	**notes**	**metal**	**contact resistivity**[Ω cm^**2**^ **]**	**refs**
Ge	2010	substrate: 1 × 10^19^; implantation of as (5 × 10^15^ cm^–2^ dose)	soak annealing at 600 °C		n-Ge/Ti	1 × 10^–3^	^59^
	2010	substrate: 1 × 10^19^		10 nm Si (doped with P ∼ 1 × 10^20^) as an intermediate layer	n-Ge/Si/Ti/TiN	1.4 × 10^–6^	^59^
	2010	substrate: 1 × 10^19^		16 nm Si (doped with P ∼ 1 × 10^20^) as an intermediate layer	n-Ge/Si/Ti/TiN	1.7 × 10^–6^	^59^
	2010	substrate: 1 × 10^19^; as as dopant	laser annealing at 900 °C	NiGe alloy at 250–330 °C	n-Ge/Ni	4 × 10^–5^	^59^
	2010	Sb as dopant ∼1 × 10^15^ cm^–2^	laser annealing	the activation level is beyond 1 × 10^20^ cm^–3^	n-Ge/Ti/Al	7 × 10^–7^	^60^
	2011	P in situ doping of 1 × 10^19^ + as implantation: 5 × 10^14^ cm^–2^	laser annealing	CVD growth at 450 °C on 200 mm Si	n-Ge/Ni	8 × 10^–7^	^61^
	2011	P in situ doping of 1 × 10^19^ + as implantation: 5 × 10^14^ cm^–2^	no thermal activation	dopant segregation during Ni germanidation (snowplow effect)	n-Ge/Ni	2 × 10^–5^	^61^
	2011	P and Sb coimplantation: ∼1 × 10^20^	rapid thermal annealing		n-Ge/Ti/Al	8 × 10^–7^	^62^
	2011	doping not specified		NiGe alloy at 300 °C	n^+^-Ge/Ni	4 × 10^–5^	^63^
	2011	doping not specified		NiGe alloy at 400 °C	n^+^-Ge/Ni	8 × 10^–5^	^63^
	2011	doping not specified		NiGe alloy at 500 °C	n^+^-Ge/Ni	6 × 10^–3^	^63^
	2012	P as dopant: 2.5 × 10^19^	postmetallization anneal at 350 °C	ZnO intermediate layer	n-Ge/n^+^-ZnO/Ti	1.4 × 10^–7^	^64^
	2014	Sb implantation: 1 × 10^20^	laser annealing		n^+^-Ge/NiGe	1.9 × 10^–8^	^65^
	2014	P and Sb coimplantation	rapid thermal annealing	active carrier concentration: ∼8.6 × 10^19^	n^+^-Ge/NiGe	6.4 × 10^–7^	^65^
	2014	Sb as a dopant: 1 × 10^20^	rapid thermal annealing	activation not specified	n^+^-Ge/NiGe	1.3 × 10^–4^	^65^
	2015	P as a dopant: ∼1 × 10^20^			n^+^-Ge/Ni	8.8 × 10^–5^	^66^
	2015	As a dopant: ∼1 × 10^20^			n^+^-Ge/Ni	1.9 × 10^–7^	^66^
	2015			activation not specified	n^+^-Ge/Ni	5.4 × 10^–7^	^66^
	2016	P as a dopant: ∼3 × 10^17^	laser spike annealing	>1 × 10^20^	n^+^-Ge/Ni	2.5 × 10^–8^	^67^
	2016	P as a dopant: ∼3 × 10^17^	laser spike annealing	activation not specified (shallow)	n^+^-Ge/Ni	2.8 × 10^–7^	^67^
	2023	P as a dopant: ∼3 × 10^20^	laser spike annealing	Ni nanoisland formation	n-Ge/Ni nanoisland/Ti	7.5 × 10^–8^	^68^
	2025	P doping 5 × 10^13^–1 × 10^14^	postmetallization at 350 °C	GeSb intermediate layer	n-Ge/GeSb/Ni	2 × 10^–2^	current work
	2025	P doping 5 × 10^13^–1 × 10^14^	postmetallization at 500 °C	GeSb intermediate layer	n-Ge/GeSb/Ni	1.2 × 10^–3^	current work

To study interfacial reactions behind the observed changes in the contact resistivity during postheating of the structures, XPS, XRD, and LEED measurements were performed. Figure 4a,b presents XPS measurements of the Sb 2p_1/2_ and Ge 3d spectra from the Sb+Ge+Sb stack on the Ge substrate as a function of the heating. The broadening of the Sb 2p_1/2_ toward the high binding-energy side arises from the incorporation of oxygen into the Sb surface during the air transfer process (about 15 min) to the XPS instrument. Also, the Sb 4d signal around 32 eV near Ge 3d in Figure 4b shows an oxide signal, shouldering around 35 eV binding energy. Upon the postheating, there is a notable reduction in the Sb core-level signals and a slight increase in the relative Ge intensity, which indicates that part of Sb has evaporated from the surface. Also, an intermixing of the Sb and Ge layers is expected to contribute to the changes in XPS intensities during the postheating. That is, part of Sb diffused toward the substrate, while part of Ge diffused toward the top during the postheating, changing the relative intensity ratio between the Sb and Ge XPS signals.

**Figure 4:**
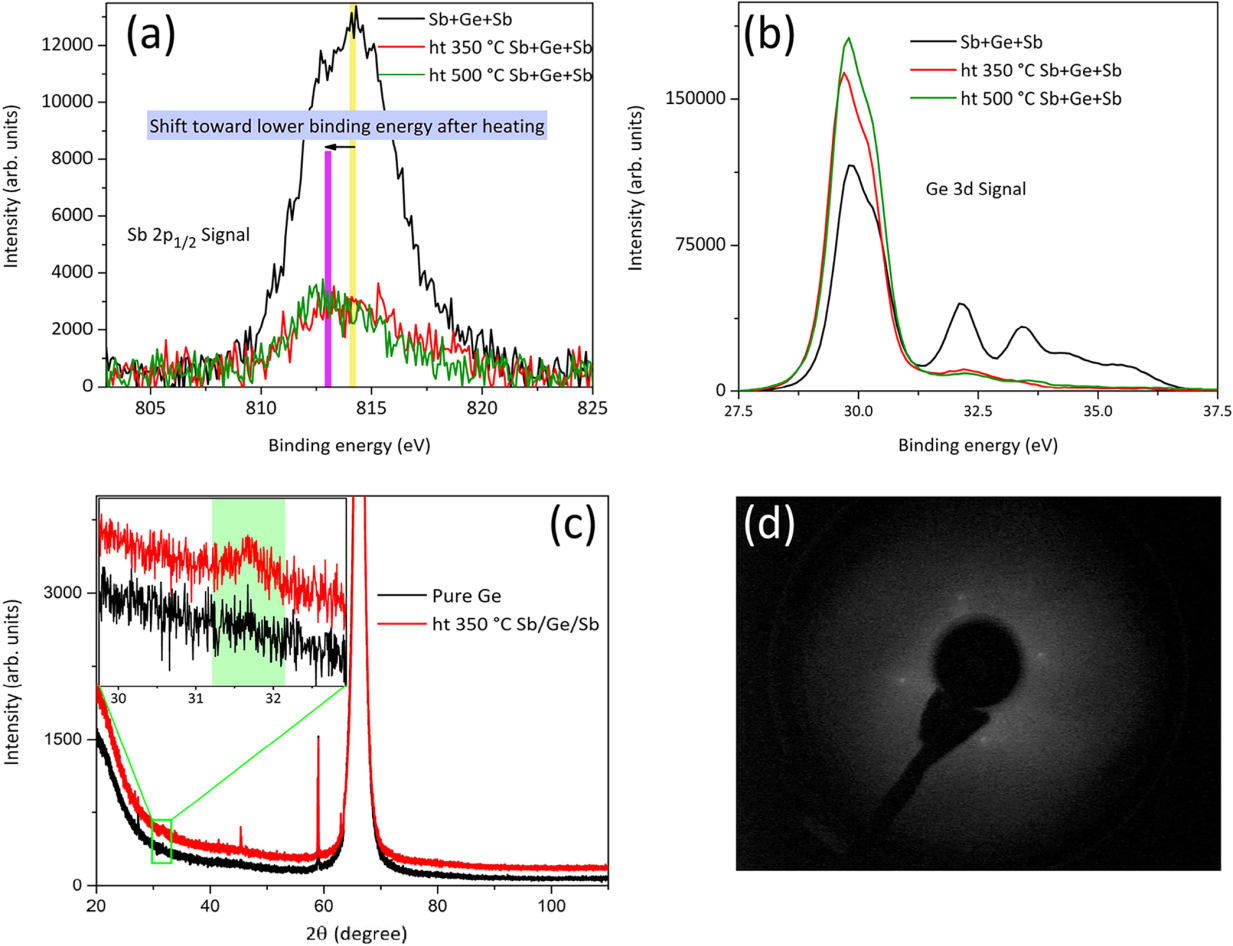
(a) and (b) XPS signals of Sb 2p _1/2_ and Ge 3d from the Sb+Ge+Sb stack on the Ge substrate, as a function of the postheating. (c) XRD patterns from the n-Ge substrate and from the sample containing a stack of Sb and Ge nanolayers. (d) LEED image after the 350 °C heating of the Sb+Ge+Sb stack on Ge shows (1 × 1) spots, indicating some crystallization in the stack.

When the Sb+Ge+Sb stack is capped by the Ni film, such diffusion-induced alloying of Ge and Sb is expected to cause the formation of a highly Sb-doped Ge interface phase(s), which is suggested to induce the Ohmic contacts in the 350 °C postheating (Sample G13, see Table 2). XRD patterns (Figure 4c) of the pure Ge substrate and the stack on n-Ge after heating at 350 °C show a small additional intensity bump around 31° for the Sb+Ge+Sb-containing sample, which might arise from Sb-rich Ge_*x*_Sb_*y*_-type crystals in the alloyed layer.^69^ The LEED pattern (Figure 4d) supports that the alloyed layer indeed has some crystalline nature (e.g., crystalline grains).

Work functions were calculated here by using the density functional theory VASP program. The work function of Ni is 4.64 eV. The work function of pure Ge is close to that of Ni. However, this Ge value is difficult to estimate exactly due to surface states that overlap with the Ge band gap area. Here, the Ge work function has been found to be 4.29–4.54 eV. If the surface atoms are replaced by Sb atoms, which leads to passivation of the Ge dimers, the band gap becomes distinct, having a value of 0.66 eV, resulting in the work function being 4.56 eV. Furthermore, if 7% of the Ge bulk atoms are replaced by Sb atoms, the work function decreases to 4.10 eV. Further Sb doping changes the work function only slightly. Therefore, it is possible that the lowered work function of the Sb-doped interface layers in our samples decreases the substrate band bending upward or even changes it downward (i.e., Ohmic type) with the accumulation of majority carriers at the substrate surface when the Fermi levels become aligned at the contacts.

### 3.3 Effects of the Sb-Doped Ge Nanofilm on the Low-Doped n-Type Si(100) Substrate

Table 4 and Figure 5 show the results for the low-doped n-Si substrates: Ni/n-Si contacts with and without an interfacial Sb-doped Ge nanofilm. The *I–V* curves of the reference Ni/n-Si contacts remained Schottky type even after the 600 °C postmetallization heating, as predicted due to the low concentration of phosphorus dopants (1 × 10^15^ cm^–3^) in the Si substrate. To increase the surface doping level beneath the Ni contacts, we used the above results observed on low-doped n-Ge as follows. We first deposited a Ge layer (4 nm) on the Si(100) substrate and then further deposited a stack of Sb (2 nm) + Ge (4 nm) + Sb (2 nm) before Ni film growth. After the postheating of this stack up to 500 °C, the *I–V* curves still showed the Schottky contact. However, the postheating around 550 °C straightened *I–V* curves toward Ohmic lines with the value of the 6.8 × 10^–2^ Ω cm^2^. In Table S2, a benchmark is presented for selected n-Si contacts. XPS Ni 2p element map and SEM-EDS measurements (Figure S4) were still performed to confirm that there was no excess of Ni remaining between the pads in our lift-off process.

**Figure 5:**
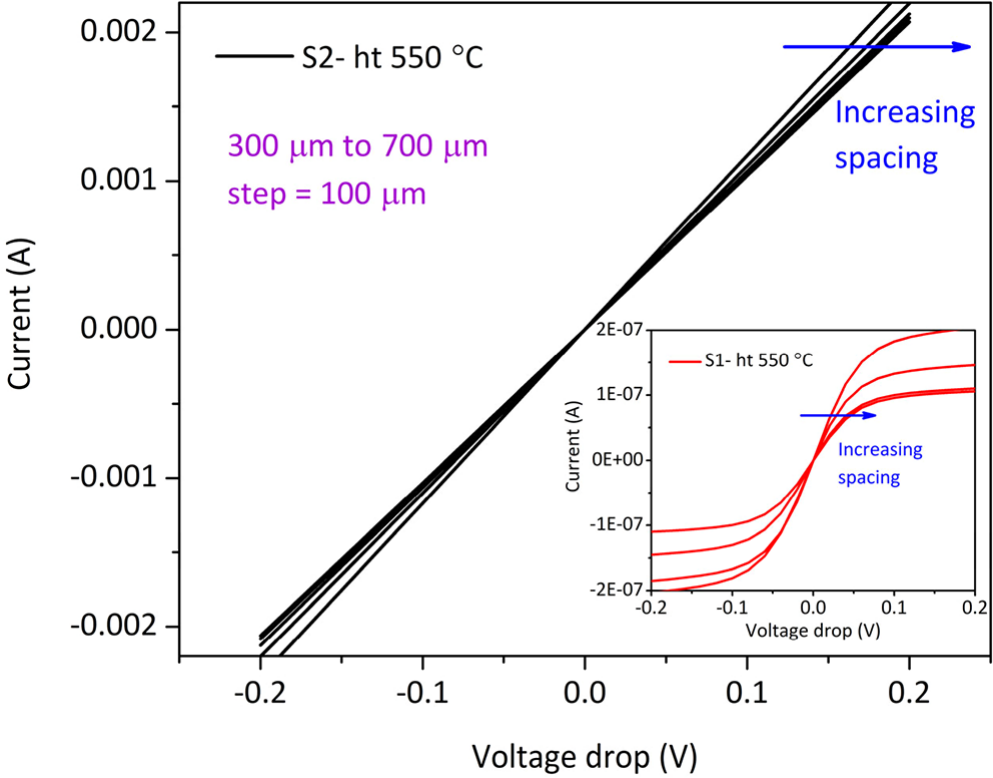
Current as a function of voltage drop for Ni/n-Si sample S2 with the intermediate Sb-doped Ge film after the postmetallization heating at 550 °C. The inset represents *I–V* for the reference Ni/n-Si sample S1 without Sb-doped Ge after the postheating at 550 °C.

**Table 4:** Properties of Ni/n-Si Contacts with and without Sb-Doped Ge^a^

									contact resistivity (Ω cm^2^)			
sample	lift-off lithography	chemical process cleaning of n-Si	Ge deposition (nm)	Sb deposition (nm)	Ge deposition (nm)	Sb deposition (nm)	Ni film thickness (nm)	photoresist remover	as-ready	postheat at 350 °C for 60 min	postheat at 550 °C for 30 min	postheat at 600 °C for 30 min
S1	√	HF (5%) for 5 s + rinsing with DW + dried with N_2_					100	acetone	Sch.	Sch.	Sch.	Sch.
S2			4	2	4	2			Sch.	Sch.	6.8 × 10^–2^	

a Lift-off processing was done on low-doped n-Si(100) substrates.

To clarify the above effects of Sb-doped Ge on Ni/n-Si contact resistivity, we characterized the Ge deposition on Si(100) as shown in Figure 6. An ultrathin 0.5 nm thick Ge still follows an initial Si(100) terrace-step structure, which formed in the flash heating of Si(100) at 1200 °C in UHV. However, smaller islands appeared at 4 nm thick Ge deposited on Si(100), indicating that a Ge film was relaxed into its own inherent lattice constant, as expected due to the large lattice-constant difference between Ge and Si. However, the 4 nm thick Ge layer still has some crystalline nature.

**Figure 6:**
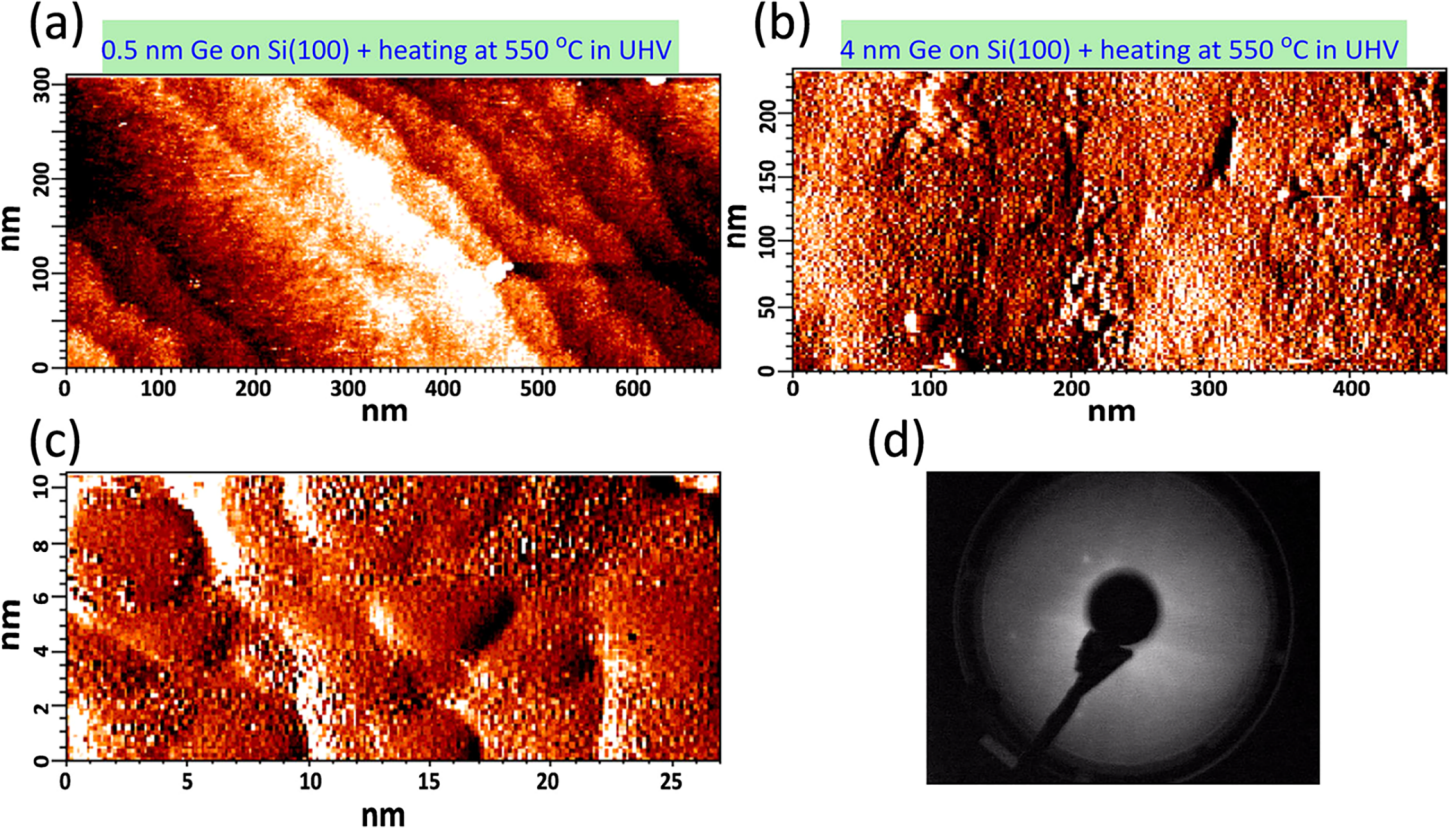
(a) STM image shows that the morphology of a 0.5 nm thick Ge layer deposited on Si(100) and postheated at 550 °C in UHV conditions follows a terrace-step structure of the initially flash-heated Si(100) substrate. (b) After depositing 4 nm of Ge and heating the stack at 550 °C, the surface contains small two-dimensional islands seen in the zoomed-in image (c). LEED (1 × 1) spots indicate that the film is at least partially crystalline; it has crystalline grains in (d).

Separate samples containing two times 10 nm thick Ge and two times 5 nm thick Sb intermediate layers at Ni/n-Si(100) were prepared for STEM measurements to study the heating-induced changes, which led to the Ohmic contacts for low-doped n-Si (Table 4). The order of deposited layers was the same as that above: Ge+Sb+Ge+Sb, providing approximately 30 nm total thickness for a modified interface before postheating. Figure 7 summarizes STEM observations alongside EDS measurements for two Ni/Sb+Ge+Sb+Ge/n-Si samples with and without the 550 °C postheating in UHV. The postheating clearly caused the interdiffusion of elements and the formation of crystalline grains in the Ni film. Such a polycrystalline nature can indeed be expected for a metal film after postheating. Because the reference Ni/n-Si sample did not provide the Ohmic contacts even after high-temperature postheating (Table 4), it is probable that the crystalline Ni grains are not the main factor behind the Ohmic-contact formation at the modified interface. In contrast, it is expected that changes near the n-Si surface are needed for the Ohmic-contact formation. Figure 7 reveals that the structure of the outermost Si surface changes during the postheating, suggesting the intermixing of Si, Ge, and Sb elements at the n-Si interface. Furthermore, local Sb-rich areas, which were seen before the postheating, disappeared after 550 °C. Similar intermixing or alloying of Ge and Sb elements was observed on low-doped n-Ge at the lower temperature of 350 °C. This is also supported by the presence of a dot-like structure near the interface, which resembles the previously observed Sb-doped Ge nanocrystals.^70^

**Figure 7:**
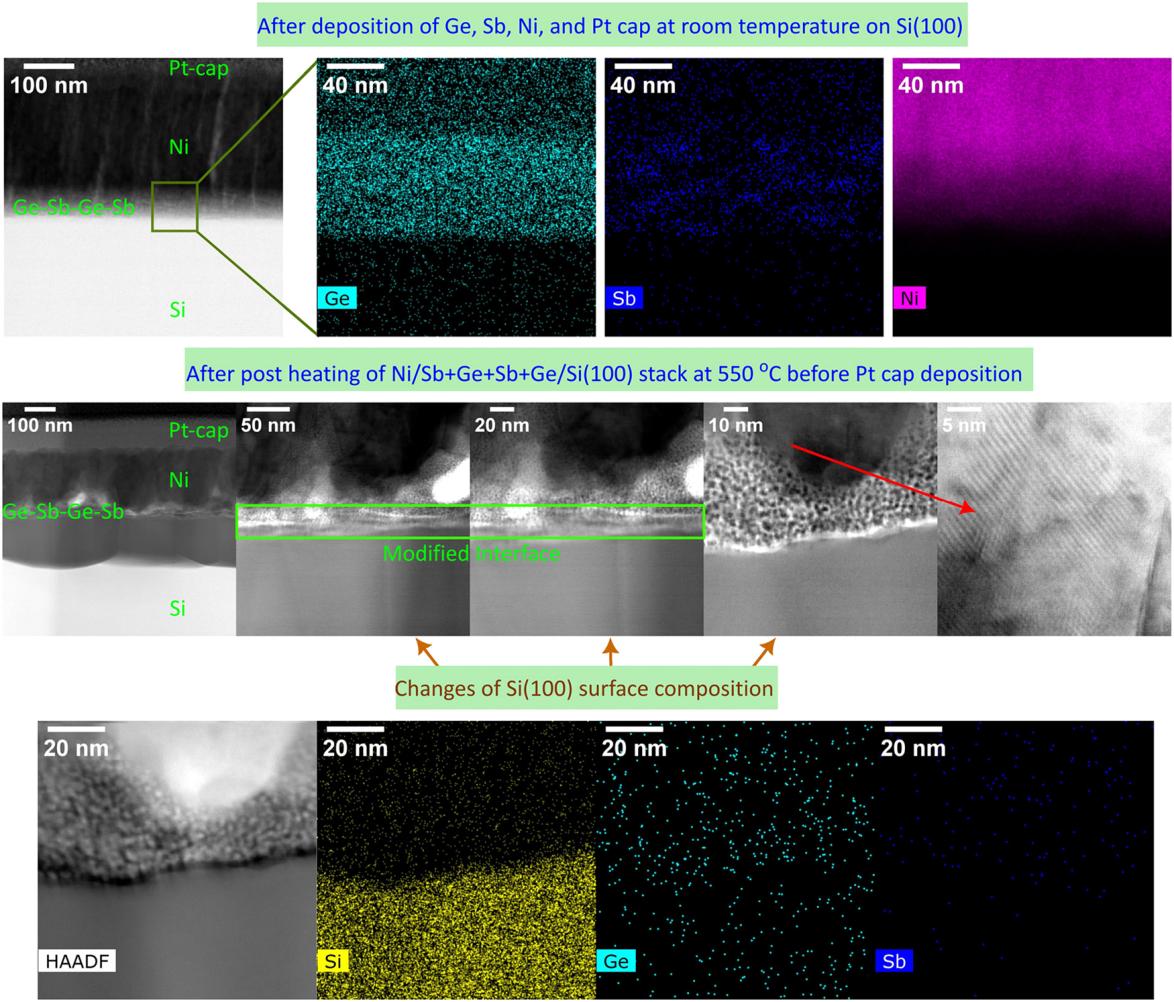
Cross-sectional STEM image alongside EDS measurements of the Ni/n-Si(100) contact interfaces, containing Sb-doped Ge, before and after postheating at 550 °C. The postheating modifies the interface, which is associated with the formation of an Ohmic contact. Before the heating, Sb-rich areas are observed, but these areas disappear due to the heating. Local crystallization is seen in the Ni metal film after the heating. Small dot-like features near the interface can arise from Sb-doped Ge nanocrystals.^70^

### 3.4 Effects of Sb-Doped Ge Nanofilm on Semi-Insulating GaAs Substrate

To evaluate the general impact of the Sb-doped Ge layer on reducing contact resistivity, we also conducted tests on the SI-GaAs substrate. Figure S5 compares the *I–V* results from Ni/SI-GaAs contacts with and without the Sb-doped Ge interface layer. Without the interface layer, the contact resistivity after 600 °C annealing was ∼1 × 10^5^ Ω cm^2^. Adding the Sb-doped Ge layer significantly decreased the resistivity to as low as 1 Ω cm^2^. However, the contact resistivity values varied in our SI-GaAs experiments from 1 to 1 × 10^3^ Ω cm^2^, likely due to uneven Ge flux prepared here using the laboratory-based evaporator. The accurate Ge deposition needs to be developed toward uniform manufacturing of devices on a large scale. Overall, ultrathin Sb-doped Ge layers decreased the contact resistivity on SI-GaAs by a factor of 100 at least.

However, future studies and development are needed to reduce the postheating temperature after the deposition of Ge and Sb layers, for example, toward different back-end-of-line processing steps. We believe that optimization of the sequence and thickness of Ge and Sb depositions, including tests of coevaporation of Ge and Sb, is useful to decrease the postheating temperature. It is also interesting to study if non-UHV conditions, like a high-vacuum environment, can be utilized to grow ultrathin Sb-doped Ge films because UHV has not been widely used in the semiconductor industry so far.

## 4 Conclusions

We demonstrated a low-temperature method to increase the n-type doping level locally at the Ge surface using Sb-doped Ge nanolayers (≤10 nm). By integrating the method with the lift-off processing of Ni contacts on the low-doped n-Ge substrate (about 1 × 10^14^ cm^–3^), we found that the Schottky contacts changed to the Ohmic ones with the contact resistivity of 2 × 10^–2^ Ω cm^2^ after the postmetallization heating at 350 °C and with the resistivity of 1.2 × 10^–3^ Ω cm^2^ after the postheating at 500 °C. The Ge substrate surface was intentionally oxidized and etched by the HCl:IPA solution to increase crystalline order at the substrate surface before depositing Ge and Sb layers in ultrahigh-vacuum conditions, keeping the substrate at room temperature. XPS and XRD measurements showed that Ge and Sb atoms intermixed and formed alloy(s) during the postheating temperature of 350 °C. XRD and LEED measurements indicated that the alloyed surface GeSb phases had some crystalline degree, which could provide local epitaxial-type interface areas on the low-doped Ge substrate, enabling Ohmic-contact formation.

Deposition of Sb-doped Ge nanolayers was also tested on the low-doped n-Si (about 1 × 10^15^ cm^–3^) substrate, which formed the Schottky contact with Ni initially. After the postheating of the Ni/n-Si contact stack that included a Sb-doped Ge interface layer around 550 °C, the Ohmic contact formed with the resistivity of 6.8 × 10^–2^ Ω cm^2^. STEM measurements showed that the postheating around 550 °C caused changes in the Si surface structure, which was associated with SiGeSb alloying at the interface. Furthermore, our preliminary tests on semi-insulating GaAs also supported that the ultrathin Sb-doped Ge films might be used on different semiconductor surfaces to modify the contact properties. Our DFT calculations of the work functions showed that if 7% of the Ge bulk atoms are replaced by Sb atoms, the work function decreases to 4.10 eV, which was suggested to decrease or remove the upward Schottky-type band bending at the different substrate surfaces.

## Supplementary Material



## Notes

Contact resistivity and XPS measurements for Ni/n-Ge samples after etching Ni film; current–voltage data after intentional oxidation of n-Ge surfaces; benchmarking table for Ni/n-Si contact resistivities; and current–voltage data for semi-insulating GaAs contacts (PDF)
