# Supplementary material for: Transforming Schottky to Ohmic Contacts via Ultrahigh-Vacuum Engineered Interfacial Alloying
Source: ACS Appl Mater Interfaces. 2025 Dec 5;17(50):68802–13. doi: 10.1021/acsami.5c21524 (PMC12723635; doi:10.1021/acsami.5c21524)
Supplement: Supplementary file 1 [file am5c21524_si_001.pdf]

Supporting Information

Transforming Schottky to Ohmic contacts via ultrahigh-vacuum engineered interfacial alloying

Masoud Ebrahimzadeh[1]*, Perttu Piispanen[1], Sari Granroth[1], Mikko Miettinen[1], Ilari Angervo[1], Hanchen Liu[2], Markus Otsus[3], Risto Punkkinen[1], Marko Punkkinen[1], Ville Vähänissi[2], Kalevi Kokko[1], Petriina Paturi[1], Kaupo Kukli[3], Hele Savin[2], Pekka Laukkanen[1]*

[1]*Department of Physics and Astronomy, University of Turku, Turku FI-20014, Finland*

[2]*Department of Electronics and Nanoengineering, Aalto University, Espoo FI-02150, Finland*

[3]*Institute of Physics, University of Tartu, Tartu EE-50411, Estonia*

* Contact: masoud.m.ebrahimzadeh@utu.fi and pekka.laukkanen@utu.fi

Table S1. Ni/n-Ge samples prepared by an approach where the Ni film first covered the whole low-doped n-Ge surface, after which part of the Ni film was chemically etched to create a contact pattern.

**Sample**	**Chemical Cleaning**	**Contact resistivity (Ωcm^2)**	
		**As-ready**	**Post heating (350 °C-60 min)**
G1	3 % HCl diluted with IPA + IPA dip (1 M)	1.56×10^{-4}	7.21×10^{-5}
G2	32 % HCl diluted with DW	1.58×10^{-4}	6.31×10^{-5}
G3	9 % HCl diluted with IPA + IPA dip (3 M)	1.86×10^{-4}	6.09×10^{-5}
G4	28 % HCl diluted with IPA + IPA dip (9 M)	6.34×10^{-5}	2.59×10^{-5}

Manufacturing Ohmic contacts is particularly challenging for low-doped semiconductors. Therefore, the measured contact resistivity values ρ_c in Table S1 are inconsistent with expectations for the n-Ge substrate with low doping concentrations (5×10^{13} to 1×10^{14} cm^{-3}). It is well known that ρ_c becomes lower with increased the surface doping concentration, as this reduces the Schottky barrier width and facilitates electron tunneling (Figure 1). Thus, the discrepancy implies that the ρ_c values in Table S1 may not properly describe the true electrical characteristics between Ni and the low-doped n-Ge substrate.

To study this issue, XPS and SEM measurements were performed, revealing the presence of $NiGe_x$ alloy between contacts, even after an extended wet chemical etching of Ni. Thus, the surface $NiGe_x$ alloy can cause an alternative parallel conduction pathway between the pads, facilitating the current flow between the contacts. To eliminate the $NiGe_x$ formation between the metal contacts, we utilized in this work the lift-off technique that is another common approach to manufacture the metal contacts for semiconductor devices.

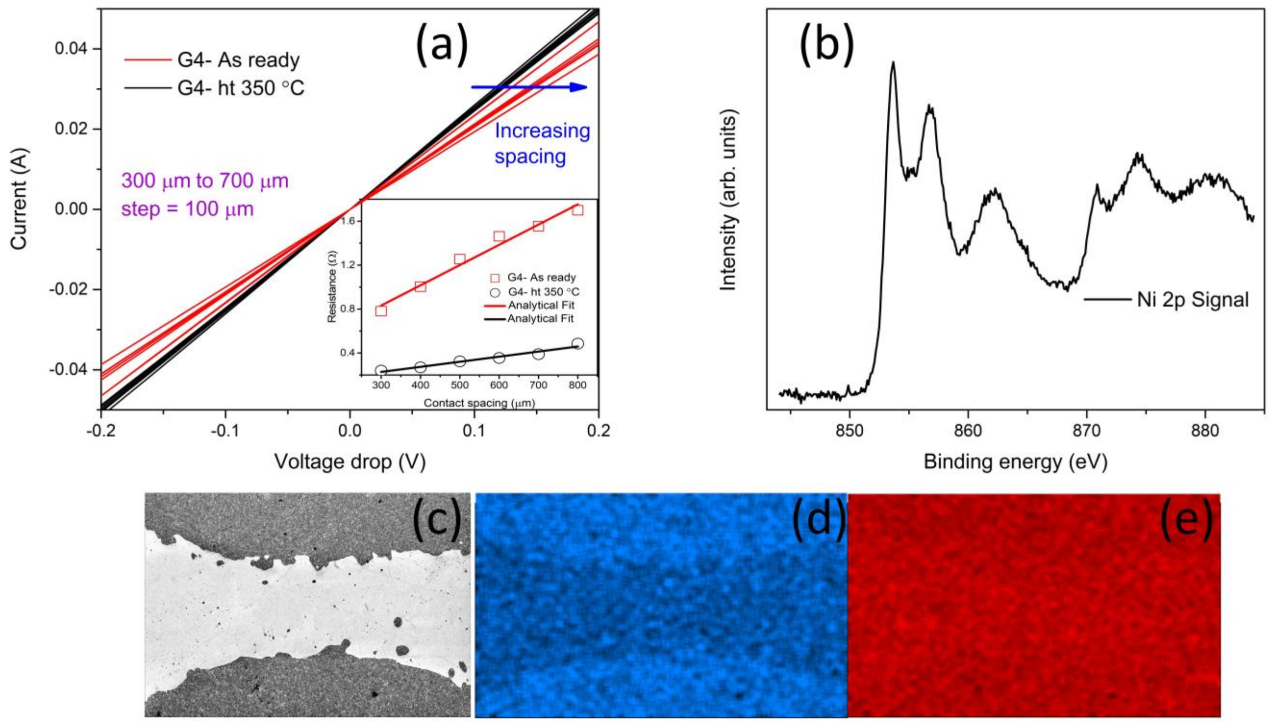

Figure S1. (a) IV plot for the sample (G4) before and after heating. The inset shows the resistance dependence as a function of the distances between pads. (b) XPS analysis of the Ni 2p signal measured between the metal pads, confirming the presence of Ni on the low-doped n-Ge surface after the extended wet chemical etching. (c), (d) and (e) SEM and EDS measurements after the etching support that Ni remained at the surface between the metal pads. The blue and red colors show the Ge and Ni elements, respectively. SEM analysis also reveals that the metal pads were not well-formed anymore.

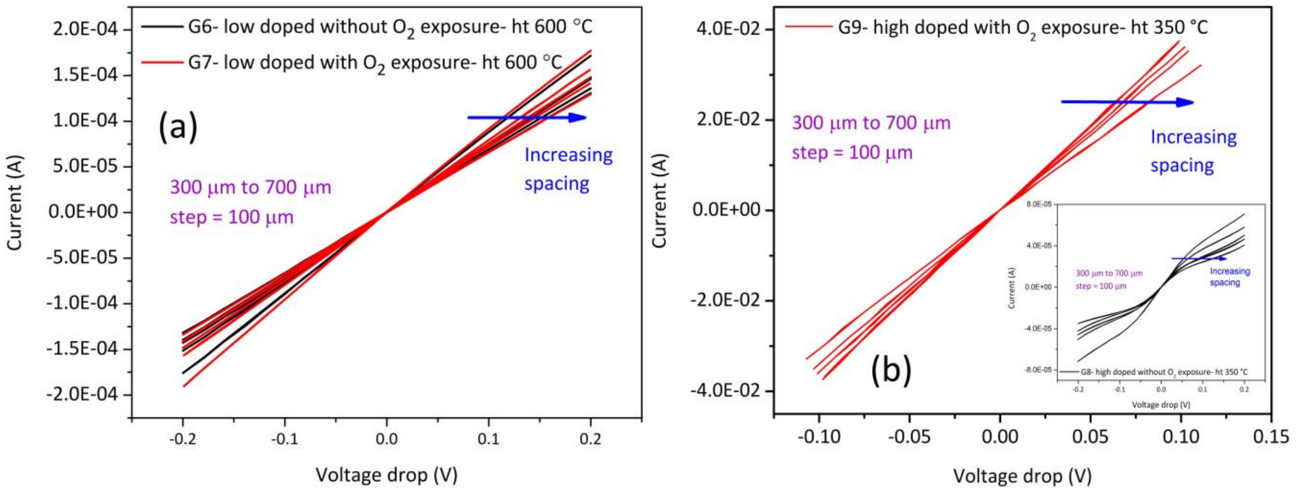

Figure S2. Current as a function of voltage drop for the samples: (a) G6 and G7, and (b) G8 (shown as an inset) and G9. The samples were exposed to O_2 gas in the UHV chamber and the HCl etched (please see the main text) before metallization and subsequently post-heated, as depicted in the plots.

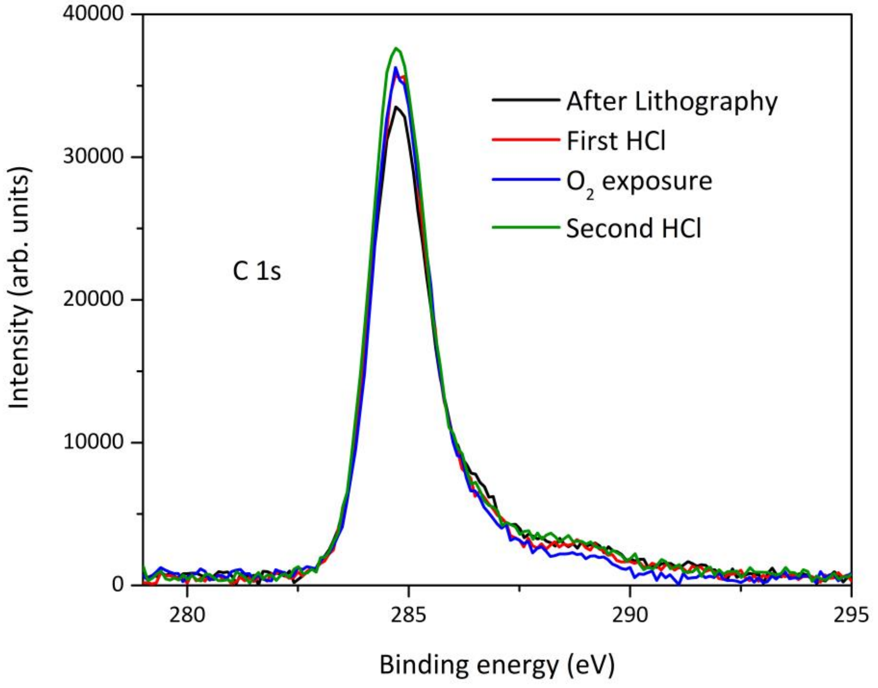

Figure S3. C 1s XPS spectra after different n-Ge surface treatments show that amount of carbon contamination did not change much.

Table S2. Comparison with previous studies on n-type Si contacts.

**Substrate**	**Year**	**Substrate doping [cm^{-3}]**	**Activation annealing**	**Notes**	**Metal**	**Contact resistivity [Ωcm^2]**	**Ref.**
Si	2012	3×10^{20}		La_2O_3 intermediate layer; Metal contact is not specified	n^+-Si/ La_2O_3/	9.5×10^{-9}	1
	2012	3×10^{20}		La_2O_3/SiO_2 intermediate layer; Metal contact is not specified	n^+-Si/ La_2O_3/ SiO_2/	2.4×10^{-8}	1
	2013	4.1×10^{20}		TiO_{2-x} intermediate layer	n^+-Si/ TiO_{2-x}/ Ti	9.1×10^{-9}	2
	2013	3×10^{20}		TiO_{2-x} intermediate layer	n^+-Si/ TiO_{2-x} / Ti	2.4×10^{-8}	2
	2014	4.1×10^{20}			n^+-Si/ Ni	1.8×10^{-6}	3
	2014	4.1×10^{20}		20 nm TiO_2 intermediate layer	n^+-Si/ TiO_2/ Ni	5×10^{-7}	3
	2015	P doping 2×10^{21}	Dynamic surface anneal	Ge preamorphization + silicidation	Si/ Ti	1.5×10^{-9}	4
	2025	P doping 5×10^{14} - 1×10^{15}	Post-metallization at 550 °C	GeSb stack	n-Si/ GeSb/ Ni	6.8×10^{-2}	Current work

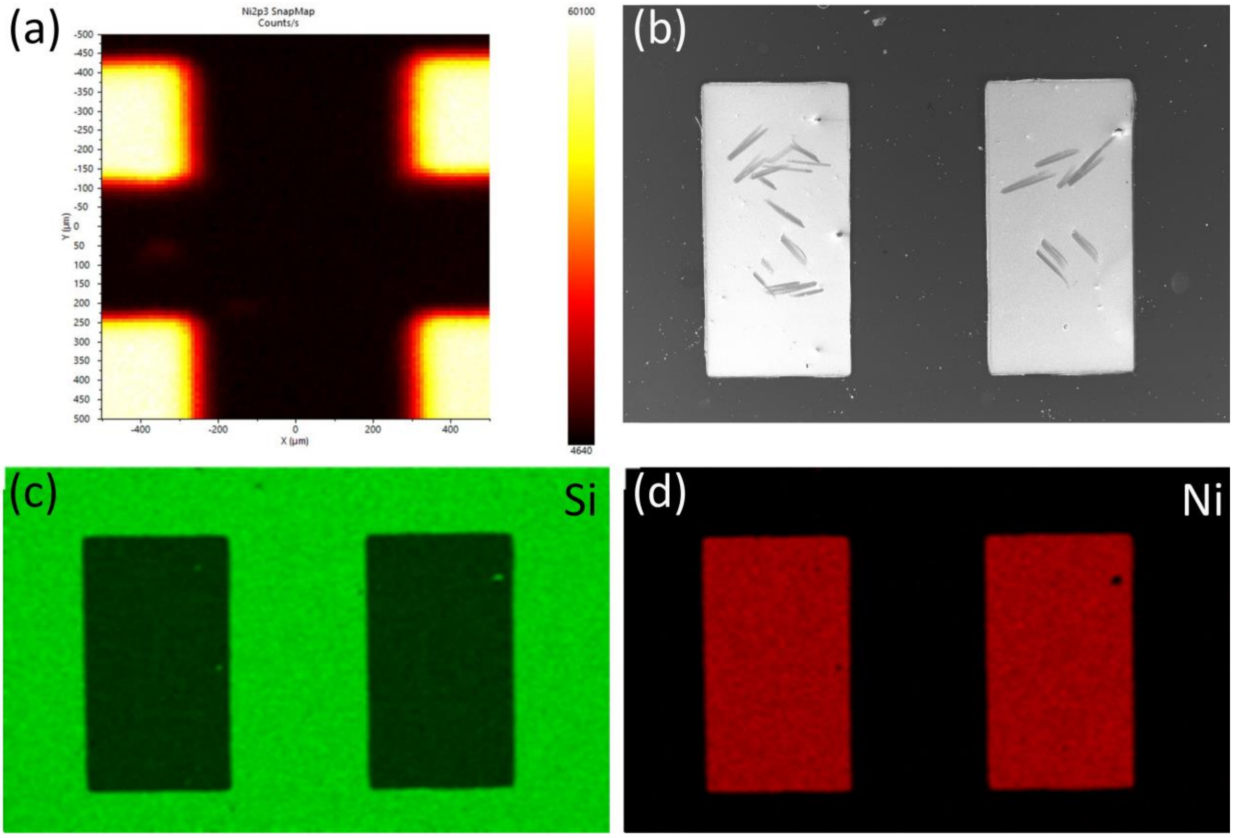

Figure S4. (a) XPS maps of the Ni 2p. (b) SEM image contact after lift-of lithography. (c) EDS mapping: Si (d) EDS mapping: Ni for the sample of S2. The scratch on the surface resulted from the 4-probe electrical measurements, where the needles made contact with the metal pads. The SEM image was taken afterward.

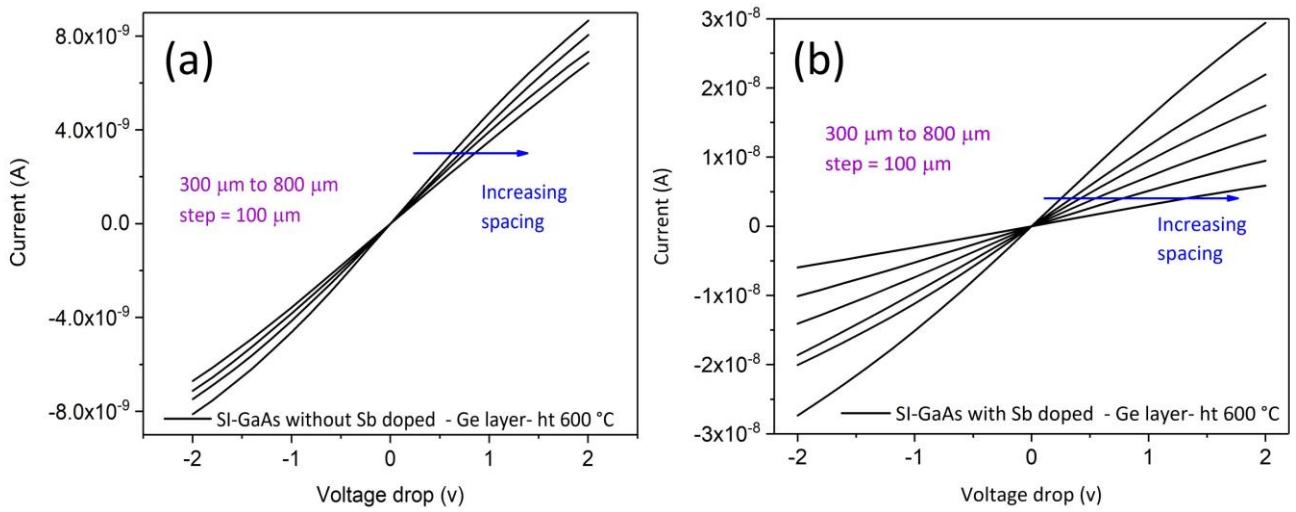

Figure S5. Current as a function of voltage drop for the Ni / semi-insulating GaAs contacts: (a) without and (b) with Sb doped-Ge layer. The samples were heated in the UHV chamber at 600 ℃ after the Ni metal deposition.

References:

(1) Ang, K. W.; Majumdar, K.; Matthews, K.; Young, C. D.; Kenney, C.; Hobbs, C.; Kirsch, P. D.; Jammy, R.; Clark, R. D.; Consiglio, S.; Tapily, K. Effective Schottky barrier height modulation using dielectric dipoles for source/drain specific contact resistivity improvement. In 2012 International Electron Devices Meeting IEEE. **2012**, 18-6.

(2) Agrawal, A.; Lin, J.; Zheng, B.; Sharma, S.; Chopra, S.; Wang, K.; Gelatos, A.; Mohney, S.; Datta, S. Barrier height reduction to 0.15 eV and contact resistivity reduction to 9.1× 10^{-9} **Ω**-cm^2 using ultrathin TiO_{2-x} interlayer between metal and silicon. In 2013 Symposium on VLSI Technology IEEE. **2013**, T200.

(3) Agrawal, A.; Lin, J.; Barth, M.; White, R.; Zheng, B.; Chopra, S.; Gupta, S.; Wang, K.; Gelatos, J.; Mohney, S. E.; Datta, S. Fermi level depinning and contact resistivity reduction using a reduced titania interlayer in n-silicon metal-insulator-semiconductor ohmic contacts. Appl. Phys. Lett. **2014**, 104, 112101.

(4) Yu, H.; Schaekers, M.; Rosseel, E.; Peter, A.; Lee, J. G.; Song, W. B.; Demuynck, S.; Chiarella, T.; Ragnarsson, J. Å.; Kubicek, S.; Everaert, J. 1.5×10^{-9} Ωcm^2 Contact resistivity on highly doped Si: P using Ge pre-amorphization and Ti silicidation. In 2015 IEEE International Electron Devices Meeting (IEDM) IEEE. **2015**, 21-7.
